# Tele-Health Intervention for Carers of Dementia Patients—A Systematic Review and Meta-Analysis of Randomized Controlled Trials

**DOI:** 10.3389/fnagi.2021.612404

**Published:** 2021-02-10

**Authors:** Aiyong Zhu, Wenting Cao, Yinghua Zhou, Anan Xie, Yun Cheng, Shu-Fen Chu

**Affiliations:** ^1^College of Nursing and Health Management, Shanghai University of Medicine and Health Sciences, Shanghai, China; ^2^Department of Nursing, Huadong Hospital Affiliated to Fudan University, Shanghai, China

**Keywords:** tele-health, intervention, carers, dementia, systematic review, meta-analysis

## Abstract

**Objective:** The purpose of this study is to evaluate the major mental health outcomes on dementia patient carers when using psychoeducational programs and psychotherapeutic interventions.

**Methods:** A meta-analysis was performed with randomized controlled trials of carers' tele-health interventions from the literature inception to December 31, 2019, using PubMed, EMBASE, and CENTRAL databases for articles.

**Results:** The meta-analysis identified 1,043 results, of which 11 were randomized control trials. Among all 11 randomized control trials, only one study addressed face-to-face contact with online modules of interventions, four studies addressed telephone-based interventions, two studies reported on combined face-to-face contact and phone call interventions, two studies focused on web-based interventions, one study used video and telephone interventions, and one study conducted a computer-telephone integration system of intervention. The updated evidence suggested that there was more efficacy via tele-health interventions in lowering depression for carers of people with dementia. We outlined the delivery formation of intervention to evaluate the effectiveness and processes of major mental health improvements, including depression, burden, anxiety, and quality of life.

**Conclusions:** In this study, tele-health intervention was shown to significantly lower depression and also lower the risk of mental health impairment. Although there was a significant decrease of depression, there were no significant differences in burden, anxiety, and quality of life. Future researchers are encouraged to carry out larger-scale studies; also, further analysis using a standardized assessment tool is suggested for future multi-component tele-health interventions.

## Introduction

Studies showed that carers of dementia family members have a poorer quality of life (QoL) (Glueckauf et al., 2007; Häusler et al., 2016; Brown et al., 2019) and higher risk for death by suicide. This risk did not decrease even after the care-recipients were institutionalized or died (Kishita et al., 2018). In 2015, there were 17.7 million informal carers who provided either in-home substantial services or unpaid assistance to their family members and friends. Though caregiving can strengthen the relationship between carer and recipient, it can also cause emotional and physical burdens on carers, resulting in higher rates of depression, lower quality of life, and poorer overall health. However, these outcomes might be eliminated when proactively addressing the needs of families and carers. To improve the support systems for carers and the care recipients, it is suggested to have a better understanding of carers and their challenges (Edwards et al., 2020). A UK survey in 2019 found that most carers (79%) used one or more types of technology, while distance carers, who do not reside in the same home with the care recipients, used slightly fewer (77%). There are lots of studies assessing the efficacy of carer interventions on emotional well-being (Orgeta et al., 2013; Wagner and Brandt, 2018; Or and Kartal, 2019). However, only a few of these reviewed the effect of intervention by delivery mode for carers of dementia patients, such as tele-health intervention. To develop more cost-effective approaches that meet the needs of people with dementia and their family carers is urgent. Different kinds of interventions could prevent or reduce the negative effect of carers, but use of internet interventions is still very limited (Blom et al., 2013). Some interventions are designed to support carers in their role, such as skills training or education to assist in caring for people living with dementia. Evidence showed that if carers learn to deal more effectively with the stresses of caregiving, their QoL will remain at a higher level. Psychoeducational interventions are the most widely studied, and improvement in level of depression and stress have been reported. Recently, there was one study that conducted a comprehensive review that suggested psychoeducation-skill building interventions show a significant influence on burden when delivered face-to-face (Kishita et al., 2018). This meta-analysis updated the literature on interventions for carers of dementia patients published between 2006 and 2016 and evaluated the efficacy of psychoeducational programs and psychotherapeutic interventions on major mental health outcomes, such as depression, anxiety, burden, and QoL. This study demonstrated strong empirical support for treating anxiety and depression by all modes of delivery (Kishita et al., 2018). Face-to-face intervention is very costly compared to the tele-health-based interventions. Tele-health interventions are much more convenient as carers can receive help when it is necessary, especially in a time such as the COVID-19 pandemic, when social distancing is crucial. Therefore, the purpose of the research is to assess the efficacy of tele-health interventions for carers of dementia patients.

## Materials and Methods

### Data Sources and Searches

Inception to December 31, 2019, we developed the search strategy, without language restriction, for PubMed, EMBASE, and CENTRAL for articles. The search terms included caregiver, carer, dementia, cognitive decline, cognitive impairment, web-based intervention, technology intervention, internet intervention, tele-health intervention, and randomized controlled trials. Titles and abstracts were screened by three reviewers independently, while full texts were sourced for relevant articles. Inclusion criteria were assessed independently, and inconsistencies were resolved by consensus. The reference lists of included trials and other published meta-analyses were also reviewed for relevant articles.

### Eligibility Criteria

According to the American Psychiatric Association's Diagnostic and Statistical Manual (DSM-5), dementia is typically diagnosed when acquired cognitive impairment has become severe enough to compromise social and/or occupational functioning. Symptoms of memory loss are caused by a range of cognitive abilities, or a general cognitive decline, and not just memory (Hugo and Ganguli, 2014). Trials were considered eligible if they were randomized clinical trials, comparing the key mental health states of anxiety, depression, and burden, and associated with poor QoL in dementia carers. Trials were required to report at least one of the following forms of intervention: blending face-to-face contact with online modules, telephone-based, combined face-to-face contact and phone calls or web-based intervention, using video and telephone interventions, or a computer-telephone integration system (Table 1). Also, trials were required to report at least one of the following outcomes: symptoms of depression, anxiety, burden, or QoL. Trials that recruited participants with face-to-face intervention only were excluded.

**Table 1:** Characteristics of included studies.

**First author**	**Year of** **publish**	**Method of** **study**	**Diagonosis of care-recipient**	**Target of population**	**Format of intervention**	**Main outcomes**
Boots L. M.	2018	RCT	People with mild dementia of all subtypes (Clinical Dementia Rating, score 0.5–1)	Family caregivers	Blending face-to-face contacts with online modules	Depression
Tremont G.	2015	RCT	Most care recipients were diagnosed with Alzheimer's disease	Family caregivers	Telephone contacts	Depression
Prick A. E.	2015	RCT	Community-dwelling people with dementia	Family caregivers	Experimental group: home visits Comparison group: information bulletins and phone calls	Depression
Cristancho-Lacroix V.	2015	RCT	Alzheimer's disease was targeted by this program	Informal caregivers	Web-based intervention	Psychologists stress
Blom M. M.	2015	RCT	People with dementia	Family caregivers	Internet course MoD	Depression.
Glueckauf R. L.	2012	RCT	People with dementia	African American dementia caregivers	Combines face-to-face and telephone-based	Depression
Gallagher-Thompson D.	2010	RCT	People with dementia	Caregivers	Using video and telephone interventions	Depressive symptoms Stress associated with memory and behavior problems
Gallagher-Thompson D.	2007	RCT	People with dementia	Female Chinese American caregivers	Telephone contacts	Depressive symptoms
Finkel S.	2007	RCT	People with Alzheimer's disease	Caregivers	Computer-Telephone Integration System (CTIS)	Depression
Winter L.	2006	RCT	People with Alzheimer's disease or related disorders	Female family caregivers	Telephone-based support groups	Depression
Burns R.	2003	RCT	People with Alzheimer's disease	Caregivers	Face-to -face and telephone contact	General Health and Mental Health

### Data Extraction

Data were collected independently by two authors using a standardized data extraction form, entered into a dedicated database, and checked independently by four authors. The data included study characteristics, baseline demographics of participants, description of the intervention, incidence of dementia, the forms of intervention, main treatment components, and length of each session (min)/treatment. We reported outcomes at the point of the longest follow-up. Eleven majority delivery modules of intervention were defined as those in which more than 50% had tele-health interventions.

### Outcomes

For our primary analysis, we used a hierarchical approach in which we included trials that reported delivery mode of intervention including blending face-to-face contact with online modules, telephone-based, combined face-to-face contact and phone calls, or web-based intervention using video and telephone interventions, or a computer-telephone integration system. We chose this approach to maximize the number of clinical trials included in our primary analysis, while also giving priority to the most tele-health intervention relevant mental health outcomes reported in trials. The main outcomes of this meta-analysis were depression, anxiety, burden, and QoL on follow-up carers of people with dementia. The outcome measures for studies were efficacy carer interventions on anxiety, depression, burden, and quality of life.

### Risk of Bias Assessment

Cochrane risk of bias tool (Higgins et al., 2011) was applied to assess the methodological quality of eligible trials on random sequence generation, allocation concealment, blinded outcome assessment, completeness of outcome data, evidence of selective reporting, and other biases. The risk of bias was independently assessed by two reviewers, while a third reviewer resolved the disagreements. A risk-of-bias summary table was created in Review Manager version 5.3. and the study was considered to be at high risk of bias if two of the domains were rated as high.

### Data Synthesis and Analysis

A descriptive analysis of each trial is provided in Table 1. Outcomes of depression, anxiety, burden, and QoL were estimated for each trial. Weighted pooled treatment effects were calculated using restricted maximum likelihood estimation to fit a random-effects meta-analysis model. A pooled mean difference and 95% CI was calculated using a random-effects meta-analysis. If this was not reported, the mean between-group difference reported at follow-up was used.

## Result

### Study Selection

**Figure 1:**
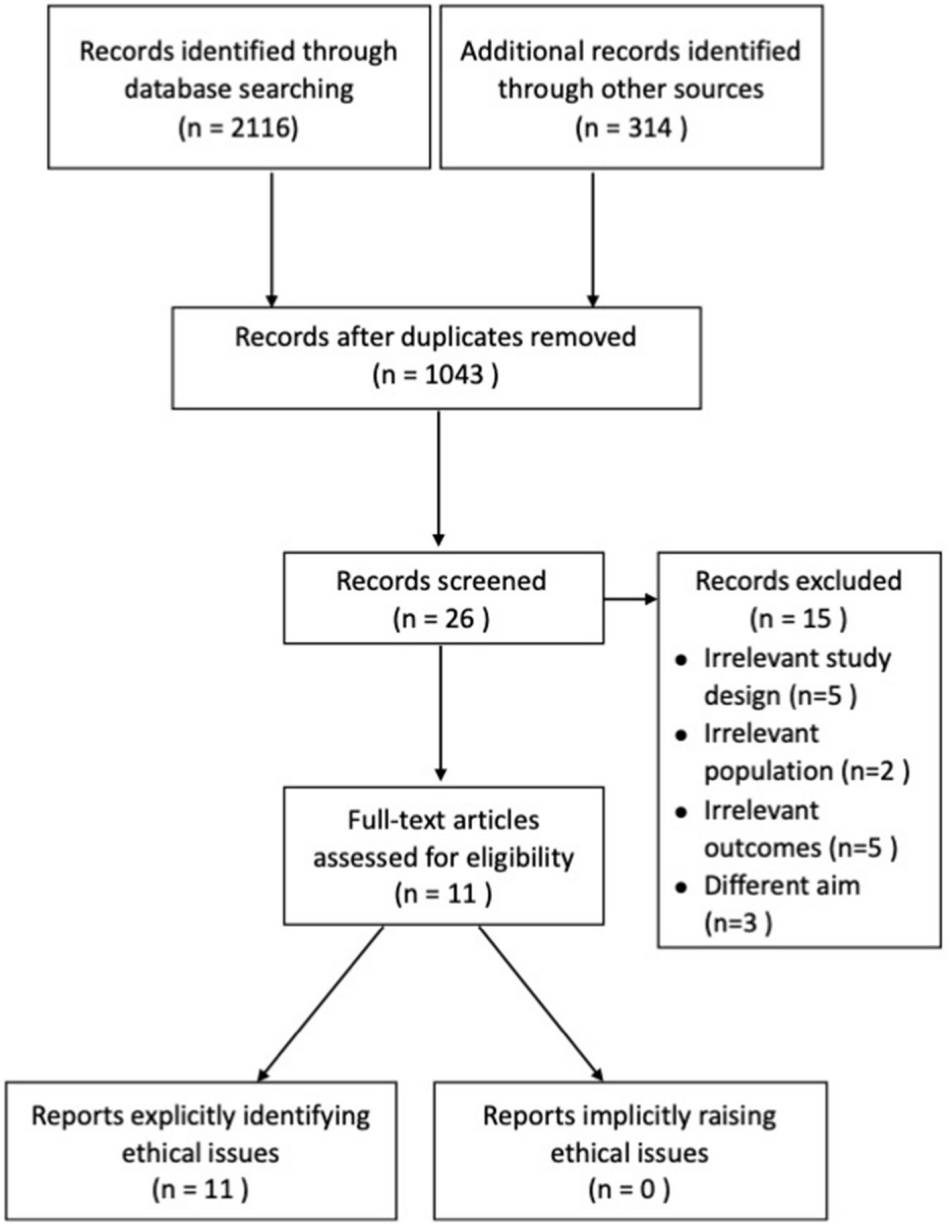
Flow diagram of the studies' selection process.

The systematic search of articles published from database inception to December 31, 2019, identified 1,043 results. After title and abstract screening, 26 articles were considered potentially relevant. Twenty studies were included after full- text review. Eleven studies reported the intervention via blending face-to-face contact with online modules (*n* = 1), four were telephone-based (*n* = 4), two combined face-to-face contacts and phone calls (*n* = 2), two used web-based intervention (*n* = 2), one used video and telephone interventions (*n* = 1), and one used a computer-telephone integration system (*n* = 1). These were included in the primary meta-analysis (Figure 1). Seven out of 11 studies were conducted in the United States (Burns et al., 2003; Winter and Gitlin, 2006; Finkel et al., 2007; Gallagher-Thompson et al., 2007, 2010; Glueckauf et al., 2012; Tremont et al., 2015), and the rest were in the Netherlands (*n* = 3) (Blom et al., 2015; Prick et al., 2015; Boots et al., 2018) and France (*n* = 1) (Cristancho-Lacroix et al., 2015). Of the 11 studies, five studies recruited carers of people with Alzheimer's disease. Most studies involved carers of people with various diagnoses of dementia grouped as a whole. In total, 1,087 participants were enrolled. The mean (SD) age of trial participants was 64.22 (5.4) years old. The publication year ranged from 2003 to 2018. Eleven trials' main outcome measurement were of depression, two trials were of anxiety, six trials were of burden, and two trials were QoL. The most commonly used measurement was the Centre for Epidemiologic Studies Depression scale (CESD). Data were available to calculate the effect size for six of the eleven studies that included burden as one of the outcome measures. The majority of studies used the Zarit Burden Interview (ZBI). Two studies reported the efficacy of interventions on anxiety. The most commonly used measure was the psychological complaints measure [Hospital Anxiety and Depression Scale-Anxiety (HADS-A)]. Two studies reported the efficacy of interventions on QoL. They used the Investigation Choice Experiments for the Preferences of Older People (ICECAP-O) and Euro-QoL to measure the efficacy.

### Study Characteristics

#### Blending Face-To-Face Contact With Online Modules and Key Mental Health

This category contained a description of only one trial on follow-up (81 participants). Carers of people with mild dementia of all subtypes (Clinical Dementia Rating, score 0.5–1) were divided into the intervention group (31 participants) and the control group (37 participants). The primary outcome was self-efficacy and symptoms of depression. Secondary outcomes included QoL and psychological complaints. The main treatment component was a blended care self-management program in combination with face-to-face coaching and tailored web-based modules. The main outcome measure for depression in this study was the CESD scale. The outcome of QoL measure was the Investigation Choice Experiments for the Preferences of Older People (ICECAP-O).

#### Telephone-Based and Key Mental Health

Four trials reported telephone-based therapy on follow-up (509 participants). Overall, 270 participants were in the intervention group and 239 participants in the control group. Symptoms of depression as primary outcomes were reported in this category. Secondary outcomes included QoL and physiological responses to stress. Two studies involved psychoeducation and skills training of communication as the main treatment component. Other studies provided problem-solving, planning of pleasant activities, and a supportive social network. The main outcome measure for depression in four studies was the CESD scale. The Dutch version of the Revised Memory and Behavior Problem Checklist (RMBPC), used self-rated general health salivary cortisol levels to measure physiological responses to stress. The outcome of the QoL measure was Euro-QoL. Self-Perceived Pressure from Family Care (SPICC) was applied to measure the planning of pleasant activities.

#### Combines Face-To-Face Contacts and Phone Calls and Key Mental Health

This category contained a description of two trials on follow-up (87 participants) and divided the carers of people with either dementia or Alzheimer's disease into the intervention group (43 participants) and the control group (44 participants). Symptoms of depression as the main outcome were reported in this category. One study included physical symptoms, psychosocial resources, and subjective burden secondary outcomes. The other study included general health and mental health and an assessment of how the dementia was manifested for the patient and affected the carer as the secondary outcomes. The main outcome measure for depression in this category was CESD scale. Modified Caregiver Health and Behavior Inventory, Assistance subscale of Interpersonal Support Evaluation List, Caregiver Appraisal Inventory, and modified General Well-Being scale were used to measured secondary outcomes.

#### Web-Based Intervention and Key Mental Health

This category contained a description of two trials on follow-up (294 participants). Overall, 174 participants were in the intervention group and 120 participants in the control group. Symptoms of depression and stress as main outcomes were reported in this category. One study included secondary outcomes such as anxiety. The other study included self-efficacy, burden, perceived health status, and depression as the secondary outcome. The CESD scale and Perceived Stress Scale (PSS-14) were the main two outcome measures for depression and stress. Hospital Anxiety and Depression Scale (HADS-A), Revised Scale for Caregiving Self-Efficacy, Zarit Burden Interview (ZBI), the French version of the Nottingham Health Profile, and Beck Depression Inventory (BDI-II) were used to measure secondary outcomes.

#### Using Video and Telephone Interventions and Key Mental Health

This category contained a description of one trial on follow-up (70 participants). Overall, 36 participants were in the intervention group and 34 participants in the control group. Symptoms of depression and stress as main outcomes were reported in this category and were measured by CESD scale and a Chinese translation of the Revised Memory and Behavior Problems Checklist (RMBPC).

#### Computer-Telephone Integration System and Key Mental Health

This category contained a description of one trial on follow-up (46 participants). Seventeen participants were in the intervention group and 19 participants were in the control group. Symptoms of depression, burden, self-care activities, and social support as outcomes were reported in this category. CESD scale, Revised Memory and Behavior Problems Checklist (RMBPC), Caregiver Health and Health Behaviors scale, and Received Social Support scale for outcomes measurement were performed.

## Risk of Bias

**Figure 2:**
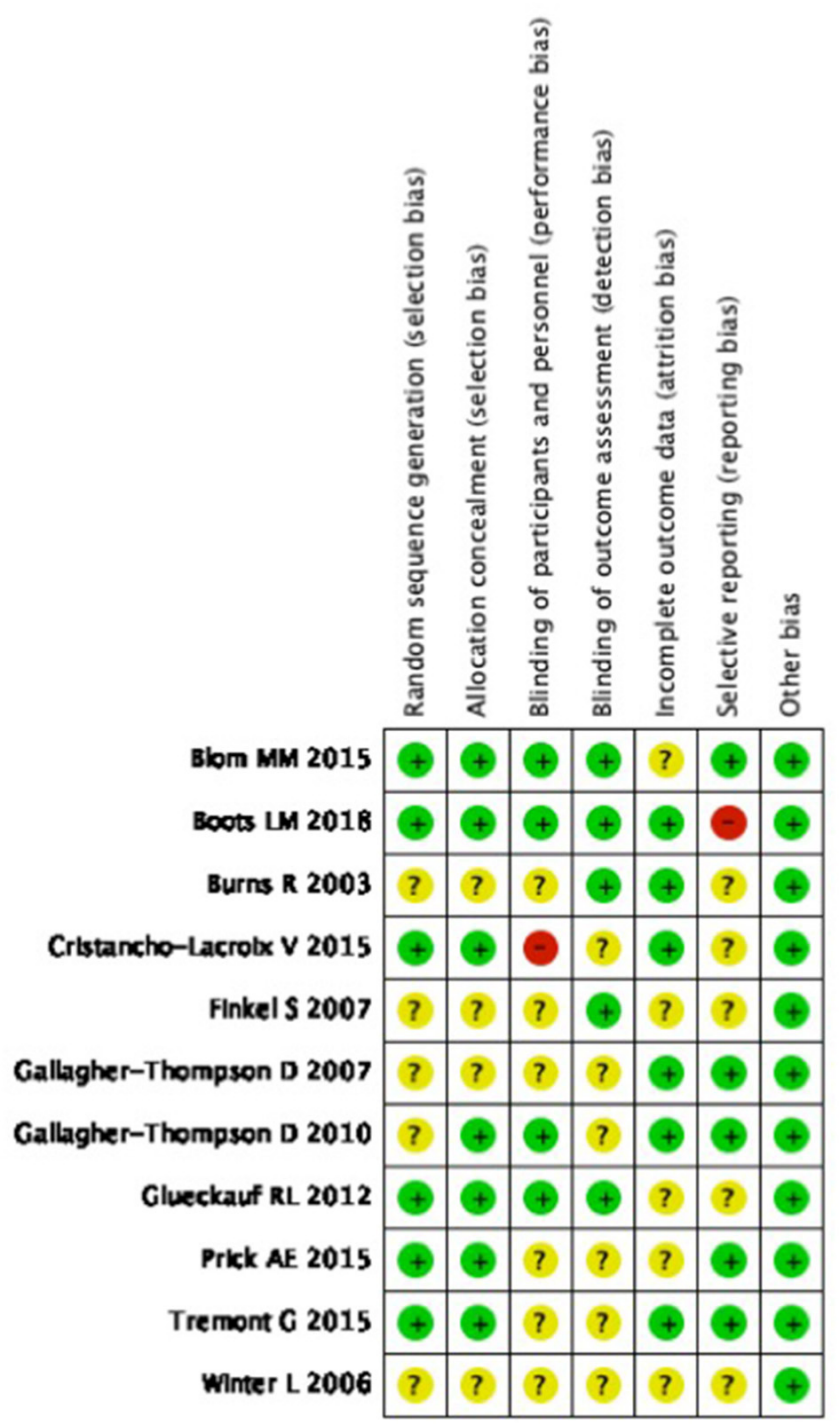
Risk of bias summary: review authors' judgements about each risk of bias item for each included study.

The quality of those studies included in our research was assessed by the Cochrane risk of bias summary and no study was excluded due to a high risk bias. All included studies were randomized trials and there were seven studies that described the specific randomization method. The majority of trials (*n* = 3) were single-blind randomized control trials. A randomization sequence was adequately generated in seven studies and 11 adequately concealed allocation. Reporting bias was noted in one trial (Figure 2). In all studies, the quality of detection bias and performance bias was relatively inconsistent, whereas the quality of publication bias was relatively low.

## Effects of Interventions

**Figure 3:**
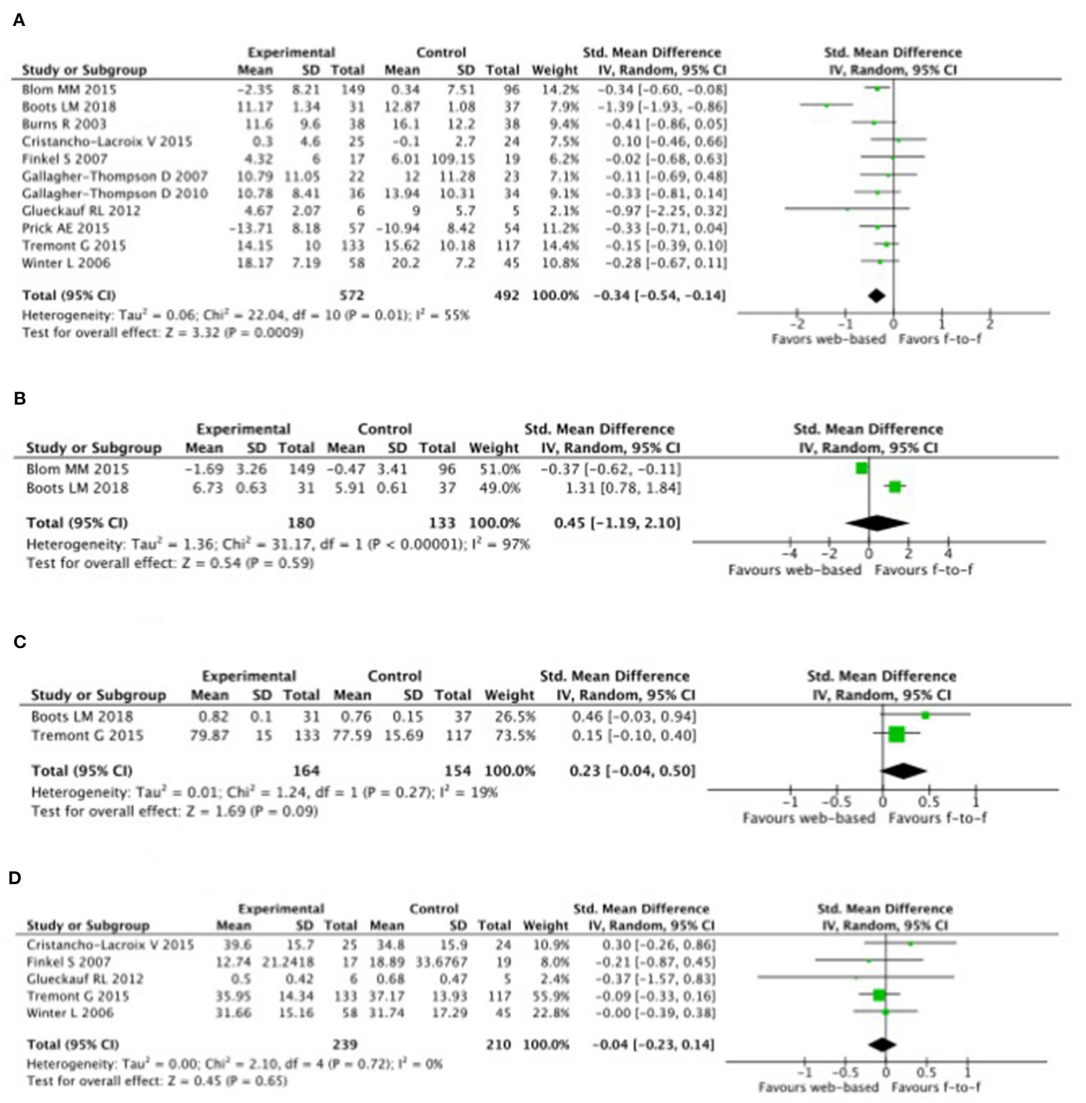
Effect sizes (Hedge's g) derived from studies examining the tele-health intervention of carers. **(A)** Depression. **(B)** Anxiety. **(C)** Quality of life. **(D)** Burden.

A random effects model was used for depression. It showed statistically significant high heterogeneity (*I*^2^ = 55%; *p* = 0.01), which had a large impact on the overall effect size (Blom et al., 2015; Tremont et al., 2015). Overall effective study size for depression was low [SMD = −0.34, *p* = 0.01; 95% CI = −0.54 to −0.14 (Figure 3A)].

Symptoms of depression with intervention formations of tele-health compared with control was highly correlated with a reduction in carers of people with dementia or AD with 7.0 vs. 7.5% over a 4.1 year-follow-up; [OR, 0.93 (95% CI, 0.88–0.98); ARR, 0.39% (95% CI, 0.09–0.68%)] (Figure 3A). Heterogeneity was low (*I*^2^ = 0.0%).

Anxiety and depression were also analyzed with a random effects model, though no heterogeneity was observed between study size. An overall effect size for anxiety was large (*g* = 2.10, *p* < 0.00001; 95% CI = −1.19 to 2.10 (Figure 3B). There was high heterogeneity between study effect sizes (*Z* = 0.54, *p* = 0.59; *I*^2^ = 97).

The efficacy of interventions on QoL and burden were evaluated with a random effects model, but the heterogeneity was not significant (*g* = 0.23, *p* = 0.27; 95% CI = −0.04 to 0.50 (Figure 3C) and the heterogeneity between study effect sizes were significantly low (*Z* = 1.69, *p* = 0.09; *I*^2^ = 19) compared with the effect size on burden, which was small [*g* = 0.04, *p* = 0.72; 95% CI = −0.23 to 0.14 (Figure 3D)].

## Discussion

Epidemiologic studies have reported a stronger association between negative states of mental health and carers of people with dementia (Phung et al., 2013). To increase carers' knowledge of the illness, develop problem-solving skills, and facilitate social support through psychoeducational interventions, interventions should be used Technology-based interventions significantly affect burden, anxiety, depression, QoL, and self-efficacy (Frias et al., 2020). Web-based interventions for carers of people with early-stage dementia demonstrated a significant improvement in self-efficacy, mastery, and QoL (Gallagher-Thompson et al., 2010). Both telephone-based and face-to-face technology-based interventions showed improvements in depression, subjective burden, and assistance support in dementia cares (Gallagher-Thompson et al., 2007). There is a significant need for non-pharmacological interventions for persons living with dementia that are easily available, easy to access, and facilitate the use of information and communication technologies (ICTs) (Gilson et al., 2019). This meta-analysis, which included 11 trials with 1,087 participants for the primary outcome analysis, found that was more efficacy via tele-health interventions in lowering depression in carers of people with dementia. We outlined the delivery formation of intervention to evaluate the effectiveness and processes of key mental health improvements, including depression, burden, anxiety, and QoL.

### Primary Findings

Our research showed that depression can be relieved more through tele-health interventions than face-to-face. Steffen et al. reported on a distance intervention carries out with a small sample size by phone and by mail that could prevent the risk of negative effects on mental health [pre-intervention score (SD), 18.9 (4.9), and post-intervention score (SD), 16.1 (3.9)] (Gant et al., 2007). There was a decrease in depression in the intervention that was associated with fewer hospitalizations at 6 months (*r* = 0.18, *P* = 0.02) (Tremont et al., 2017). The current study found that carers in the tele-health intervention group had less case-level depression [odds ratio (OR) 0.24, 95% CI 0.07–0.76] (Livingston et al., 2014). This was also proven in Zhao et al.'s study in which depression scores dropped an average of 0.23 (95% CI −0.38 to −0.07; *P* < 0.01) after web-based interventions (Zhao et al., 2019); otherwise, in the study by Kajiyama et al. (2013), it showed evidence-based interventions for carers via the internet for reducing stress was significant (*p* = 0.017) but no significant changes in the bother, depression, or level of life quality. There was no significant effect on depression that was statistically significant effected by Time (*p* = 0.27), Group (*p* = 0.53), Ethnicity (*p* = 0.34), the Group ^*^ Time ^*^ interaction (*p* = 0.92), or the Group ^*^ Ethnicity ^*^ Time ^*^ interaction (*p* = 0.08) (Czaja et al., 2013). A cross-sectional study reported that carers of AD dementia patients frequently experienced hypertension (12.7%) and insomnia (11.0%) but there were no significant differences on depression and other comorbidities (Montgomery et al., 2018). However, the tele-health intervention should be an important measure to help carers of dementia in the future, especially in conjunction with the fast improvement of associated technologies.

### Secondary Findings

One report by Austrom et al. (2015) showed that a weekly web-based video conference support group for carers could relieve their anxiety (mean difference 1.5, improvement 75%) and burden (mean difference −1.0, improvement 50%). Tsolaki et al. (2015) found ICT can not only help patients with cognitive, functional, and behavioral problems, but also support health professionals and carers by reducing their anxiety compared with face-to-face interventions. There is also a meta-analysis that found that web-based interventions significantly improved the anxiety status of carers by −0.32 (95% CI −0.50 to −0.14; *P* = 0.0005) (Zhao et al., 2019). However, McKechnie et al. (2014) reported no change in depression or anxiety over the 12-week study period via online peer support. In one study, carers who had access to a website improved their confidence while role strain, anxiety, and depression were decreased, although some of them were not significant (Zimmerman et al., 2018). However, a small sample size, low test efficiency, and a high rate of loss to follow-up over time limits this study and further research is needed to draw a conclusion.

#### Burden

Our study showed that the mental health burden would be reduced through tele-health intervention. Some studies showed a correlation between scores on depression, anxiety, and sleep quality assessments and carers' burden (Coffman et al., 2017). Online contact with a professional led to relief in burden and strain for those carers that appreciated personalized practical advice and emotional support (Hopwood et al., 2018). However, not many studies support this result. Parker et al. (2008) performed a meta-analysis study that indicated that there were no significant effects from any of the interventions. Therefore, the major outcome for web-based learning courses was the increase in knowledge. No effects were found on the relief of burden (Hattink et al., 2015).

#### Quality of Life

Our study revealed that tele-health interventions could improve quality of life for carers of dementia patients.

Although there was one study that indicated that quality of life was only moderate (SD = 8.21) and negatively correlated with burden (*r* = −32, *p* = 0.01) and depression (*r* = −0.296, *p* < 0.05) (Papastavrou et al., 2014), the findings from Bartfay and Bartfay (2013) showed similar levels of QOL between carers who used community-based interventions. Another systemic meta-analysis showed the effectiveness of IT interventions in the carers of elderly dementia both improving the quality of life and effects on behavior (such as less falling) (Nijhof et al., 2009). However, Duggleby et al. (2018) reported no significant differences from web-based interventions on health-related quality of life, neither for carers of community-living persons with Alzheimer's disease, nor for related dementia.

### Quality of the Included Studies

All results of the quality assessment for the included studies were rated as medium, which explained the variation in many domains, such as theoretical model selection, intervention format, and evaluation tools. It also increased the difficulty in assessing the risk of bias in many areas, which resulted in inconsistent results. There is more and more evidence proving the feasibility of tele-health multicomponent interventions and suggesting the most useful and least expensive intervention components or platforms. Further research on carer-focused interventions to improve their mental health is suggested in the future.

## Limitations

There are a few limitations that may lessen the credibility of our study. First, the study was not assessed by a standardized tool, and only limited variables were analyzed. Second, only the USA, Dutch, and France were included, which may limit the ability to conclude the result in other nationalities. Third, there were large differences in the usage of assistive technology, outcome measures, and the quality of studies. All of these created difficulties in comparing results across reviews.

## Conclusions

In this meta-analysis of randomized clinical trials, depression lowering with tele-health interventions compared with a controlled group was significantly associated with a lower risk of incident mental health impairment. Although there was a significant decrease in depression, there were no significant differences in burden, anxiety, or quality of life. More rigorous methods and larger-scale, higher-quality studies and multicomponent tele-health interventions are suggested in the future to analyze the outcome in further detail using a standardized assessment tool.

## Data Availability Statement

The raw data supporting the conclusions of this article will be made available by the authors, without undue reservation.

## Author Contributions

All authors listed have made a substantial, direct and intellectual contribution to the work, and approved it for publication.

## Conflict of Interest

The authors declare that the research was conducted in the absence of any commercial or financial relationships that could be construed as a potential conflict of interest.
